# Single-cell and multi-omics analysis identifies TRIM9 as a key ubiquitination regulator in pancreatic cancer

**DOI:** 10.3389/fimmu.2025.1631708

**Published:** 2025-09-19

**Authors:** Liang Chen, Xiaomei Ying, Chenfeng Ma, Qikai Tang, Shuai Chen

**Affiliations:** ^1^ Department of Hepatobiliary and Pancreatic Surgery, Conversion Therapy Center for Hepatobiliary and Pancreatic Tumors, First Hospital of Jiaxing, Affiliated Hospital of Jiaxing University, Jiaxing, Zhejiang, China; ^2^ Department of General Surgery, Suzhou Hospital of Anhui Medical University, Suzhou, Anhui, China; ^3^ Department of Neurosurgery, Nanjing Jinling Hospital, Affiliated Hospital of Medical School, Nanjing University, Nanjing, Jiangsu, China

**Keywords:** ubiquitination, pancreatic cancer, single-cell RNA sequencing, TRIM9, HNRNPU

## Abstract

This study investigates the role of ubiquitination-related genes in pancreatic cancer (PC) using single-cell RNA sequencing (scRNA-seq), spatial transcriptomics, and multi-omics approaches. scRNA-seq data (GSE155698) from PC samples identified 12 cell types, with endothelial cells exhibiting high ubiquitination scores (High_ubiquitin-Endo) and enriched interactions with fibroblasts/macrophages via WNT, NOTCH, and integrin pathways. Spatial transcriptomics (GSE235315) validated cell-type localization. Mendelian randomization (SMR) analysis prioritized TRIM9 as a PC-protective gene, downregulated in tumors and correlated with better survival. WGCNA revealed TRIM9-co-expressed modules linked to prognosis. A machine learning-based prognostic model (CoxBoost+RSF) integrating seven genes (TSPAN6, TSC1, RNF167, PBXIP1, LRRC49, KATNAL2, IGF2BP2) stratified patients into high/low-risk groups with distinct survival, mutation burdens, and immune infiltration. TRIM9 overexpression suppressed PC cell proliferation/migration *in vitro*, while knockdown enhanced malignancy. Mechanistically, TRIM9 promoted K11-linked ubiquitination and proteasomal degradation of HNRNPU, dependent on its RING domain. *In vivo*, TRIM9 overexpression reduced tumor growth, rescued by HNRNPU co-expression. Integrated analyses highlight TRIM9 as a tumor suppressor and prognostic biomarker, mediated via ubiquitination-dependent regulation of HNRNPU stability. This work provides insights into ubiquitination-driven PC pathogenesis and therapeutic targeting.

## Introduction

Pancreatic cancer is one of the most aggressive and challenging malignancies, characterized by late-stage diagnosis, poor prognosis, and resistance to available therapies (1–3). Despite advances in cancer research, the molecular mechanisms driving pancreatic cancer progression remain poorly understood, highlighting the need for deeper insights into its cellular and molecular landscape (4, 5). Among the many regulatory mechanisms implicated in tumorigenesis, post-translational modifications, particularly ubiquitination, have gained significant attention due to their critical role in controlling protein stability, degradation, and cellular signaling pathways (6).

Ubiquitination is a reversible process in which ubiquitin molecules are covalently attached to target proteins, regulating their fate within the cell (7). This modification is essential for many cellular processes, including protein turnover, cell cycle regulation, and stress responses, all of which are crucial in cancer development (8). Ubiquitination-related pathways have been shown to influence key regulatory proteins, such as oncogenes, tumor suppressors, and transcription factors (9). However, the specific role of ubiquitination in regulating proteins involved in pancreatic cancer, particularly the mechanisms that control the stability of these proteins, remains an area of active investigation (10).

In this study, we first conducted a comprehensive bioinformatics analysis of ubiquitination-related genes using multiple pancreatic cancer datasets. By analyzing gene expression patterns and identifying genes associated with ubiquitination, we uncovered several candidates that could potentially regulate cancer-related pathways. Among these, TRIM9 (tripartite motif containing 9) emerged as a key gene of interest. TRIM9, a member of the TRIM family, has been implicated in various cellular processes, including immune response regulation, protein degradation, and cell signaling (11). However, its role in pancreatic cancer, particularly in regulating the stability of critical tumor-associated proteins, had not been thoroughly explored prior to this study.

Our subsequent investigation revealed that TRIM9 may regulate the stability of HNRNPU (heterogeneous nuclear ribonucleoprotein U), a multifunctional RNA-binding protein involved in RNA processing and cell survival (12). Previous studies have suggested that HNRNPU plays a role in promoting tumor progression, yet the mechanisms by which its stability is controlled, particularly in the context of pancreatic cancer, remain unclear.

To test this hypothesis, we employed a variety of computational and experimental approaches, including single-cell RNA sequencing (scRNA-seq), spatial transcriptomics, and transcriptomic data analysis, to explore the expression patterns of TRIM9 and HNRNPU in pancreatic cancer. Additionally, we integrated ubiquitination-related gene sets and performed gene co-expression analyses to identify potential signaling pathways regulated by TRIM9. Furthermore, we used GWAS (genome-wide association studies) and SMR (Summary-data-based Mendelian Randomization) analyses to examine the genetic associations between TRIM9 and pancreatic cancer susceptibility.

By examining the ubiquitination-dependent regulation of TRIM9 and its potential impact on HNRNPU stability, this study contributes to the growing body of knowledge on the molecular mechanisms underlying pancreatic cancer. Our findings offer novel insights into the role of ubiquitination in tumor progression and provide a foundation for future therapeutic strategies targeting ubiquitin-related pathways in pancreatic cancer.

## Materials and methods

### Single-cell data processing

Single-cell RNA sequencing data related to pancreatic cancer (GSE155698) were downloaded from the GEO database (13). This dataset includes 17 tumor samples. After quality control, the samples in the standard 10X format were retained, resulting in 16 samples for further analysis. The “Seurat” package (version 4.4.0) was used for processing and analyzing single-cell data. The quality control criteria were as follows: 1) genes expressed in fewer than 3 cells were excluded; 2) cells with fewer than 200 or more than 7000 genes expressed were removed; 3) cells with more than 15% mitochondrial gene expression were excluded; 4) cells with total gene expression above 50,000 were removed; 5) samples with fewer than 100 cells were discarded. The “NormalizeData” function was used for data normalization. Highly variable genes were selected with a cutoff of 3000, using the “vst” method. The “CCA” method was applied for sample integration. “PCA” analysis was first conducted to reduce the dimensionality of the data. “tSNE” and “UMAP” methods were then applied to further reduce dimensionality, with the setting of dims = 20. “KNN” clustering was performed using dims = 20, resolution = 0.6, and random.seed = 2024. Cells were annotated based on known marker genes for pancreatic cancer cell types. UMAP plots were used to visualize the single-cell analysis results. Differential expression analysis between two groups was performed using the “FindMarkers” function from the “Seurat” package. Gene set enrichment scores were quantified using the “irGSEA,” “AUCell,” and “UCell” packages. Cell-cell communication was analyzed using the “CellChat” package to explore the interactions and receptor-ligand expression patterns between different cell types.

### Spatial transcriptomics data acquisition and processing

Spatial transcriptomics data for pancreatic cancer (GSE235315) were downloaded from the GEO database, and three samples were selected for further analysis (14). The “Seurat” package was used for data processing, with the “LogNormalize” method for normalization. Dimensionality reduction was performed using “PCA” with dims = 30, followed by clustering with the “FindClusters” method. To map the cell types from single-cell analysis to the spatial transcriptomics data, we employed the “spacexr” package’s RCTD deconvolution method, which allowed us to annotate tissue types in spatial transcriptomics data. The results were visualized using the “SpatialFeaturePlot” function.

### Transcriptomics data processing

Multiple pancreatic cancer cohort datasets containing both transcriptomics and clinical data were downloaded. The TCGA dataset was obtained from the UCSC Xena website (https://xenabrowser.net/), and the AU and CA cohort data were downloaded from the ICGC website (https://dcc.icgc.org/). Additional datasets (GSE28735, GSE57495, GSE62452, GSE78229, GSE79668, GSE85916) were retrieved from the GEO database (15–19). All data were normalized. Samples containing both transcriptomic and clinical data were retained by matching these two datasets.

### Acquisition of ubiquitination-related gene set

Ubiquitination-related genes were obtained from the GeneCard database, using a relevance score greater than 10, resulting in a list of 405 genes associated with ubiquitination.

### GWAS data download for pancreatic cancer

Pancreatic cancer GWAS datasets, including bbj-a-140 and ebi-a-GCST90018673, were downloaded from the IEU database (https://gwas.mrcieu.ac.uk/).

### SMR analysis

SMR analysis was performed to identify genes associated with pancreatic cancer susceptibility. The “TwoSampleMR” R package (version 0.6.6) and SMR software were used for analysis, with the following parameters: p_SMR < 0.05 and P_HEIDI >0.05. Results were visualized using the “SMRLocusPlot” and “SMREffectPlot” functions.

### WGCNA analysis

WGCNA (Weighted Gene Co-expression Network Analysis) was used to identify gene modules associated with pancreatic cancer traits. In this study, WGCNA was employed to explore genes co-expressed with TRIM9 in pancreatic cancer. The soft-thresholding power was set to integers between 1 and 20, with a step size of 1. The “WGCNA” package’s “pickSoftThreshold” function was used to determine the optimal soft-thresholding power. A minimum module gene size of 200 was set, with deepSplit = 2, followed by gene clustering. The resulting modules were correlated with TRIM9 and survival data.

### GO analysis

Gene Ontology (GO) is a database established by the Gene Ontology Consortium to provide a structured and standardized vocabulary to describe the functions of genes and proteins across species. It is divided into three categories: Biological Process (BP), Cellular Component (CC), and Molecular Function (MF).

### KEGG pathway analysis

The KEGG (Kyoto Encyclopedia of Genes and Genomes) database provides a comprehensive analysis of gene functions and links genomic information to functional pathways, including metabolic pathways, gene families, and cellular pathways. Gene enrichment analysis was performed by comparing the studied genes to the KEGG pathways.

### Construction and validation of prognostic model

In the TCGA cohort, univariate Cox regression analysis was first performed to identify prognostic genes with a significance threshold of p < 0.05. Prognostic models were then constructed using different machine learning methods such as RSF, GBM, CoxBoost, and LASSO (20–23). Patients were divided into high-risk and low-risk groups based on the median risk score. Survival curves, ROC analysis, and PCA were performed for both groups, and the model was validated in other cohorts.

### Copy number variation and tumor mutation analysis

The “maftools” package was used to download mutation data for pancreatic cancer. Samples containing both transcriptomic and mutation data were retained for further analysis, and mutation landscapes were visualized using a waterfall plot. Copy number variation analysis was conducted using the GISTIC_2.0 module from GenePattern (https://cloud.genepattern.org/) and visualized using the “ggplot2” package and heatmaps.

### Immune microenvironment analysis

Immune cell infiltration data for the TCGA dataset were obtained from the TIMER2 website (http://timer.cistrome.org/), and results were visualized using a waterfall plot. The “estimate” package was used to calculate immune scores for each sample. The TIDE website was used to predict immune therapy responses and TIDE scores.

### Clinical tissue sample acquisition

Pancreatic cancer clinical tissue samples were obtained from patients diagnosed with pancreatic cancer who underwent surgery at Jiaxing First Hospital. Written informed consent was obtained from all patients prior to sample collection, in accordance with the institutional ethical guidelines and the Declaration of Helsinki. Tumor tissue and adjacent normal tissue samples were collected during surgery, immediately frozen in liquid nitrogen, and stored at -80°C until further use. The samples were confirmed by pathologists to ensure they were tumor tissues or matched normal tissues.

### Cell culture

The human pancreatic cell lines HPDE6-C7, Panc-1, Capan-1, Spc-1, and MIA PaCa-2 were cultured under standard conditions. HPDE6-C7, an immortalized normal pancreatic duct epithelial cell line, was maintained in Keratinocyte Serum-Free Medium (K-SFM) supplemented with epidermal growth factor (EGF) and bovine pituitary extract (BPE). Panc-1, Capan-1, Spc-1, and MIA PaCa-2, all human pancreatic cancer cell lines, were cultured in Dulbecco’s Modified Eagle Medium (DMEM) supplemented with 10% fetal bovine serum (FBS) and 1% penicillin-streptomycin. All cells were maintained in a humidified incubator at 37°C with 5% CO_2_ and subcultured at 80-90% confluence using 0.25% trypsin-EDTA.

### Plasmid and siRNA transfection methodology

Plasmids including Flag-TRIM9-WT, Flag-TRIM9△RING, Myc-HNRNPU, HA-Ub-WT, and ubiquitin mutants HA-Ub-K6, K11, K27, K48, K63 were transfected into cultured cells using Lipofectamine 3000 (Invitrogen, #L3000015) according to the manufacturer’s instructions. For overexpression of TRIM9, the Flag-TRIM9-WT plasmid was transfected alone or co-transfected with HA-Ub plasmids to investigate ubiquitination modifications. In addition, two TRIM9-specific siRNAs (siTRIM9–1 and siTRIM9-2) and a negative control siRNA (si-NC) were transfected using Lipofectamine RNAiMAX (Invitroge) following standard protocols. For all transfections, cells were seeded in 6-well plates and allowed to reach approximately 70-80% confluence. Plasmids (2-4 µg) or siRNA (50–100 nM) were diluted in Opti-MEM medium (Gibco, #31985062) and mixed with transfection reagents. The mixtures were incubated at room temperature for 15 minutes before being added to the cells. After 24–48 hours of transfection, cells were harvested for subsequent experiments, including protein expression analysis, co-immunoprecipitation (Co-IP), and ubiquitination assays.

### Cycloheximide and MG132 treatment

To assess protein stability, cells were treated with cycloheximide (CHX, Sigma-Aldrich, #C7698) at a final concentration of 100 µg/mL for various durations (0, 2, 4, and 6 hours) prior to protein extraction. This treatment was used to block *de novo* protein synthesis and monitor protein degradation over time.

For proteasome inhibition assays, cells were treated with MG132 (Selleck, #S2619) at a concentration of 10 µM for 6 hours before harvest to evaluate the role of the ubiquitin-proteasome pathway in regulating protein degradation. All drug treatments were performed in HEK293T and PANC-1 cell lines unless otherwise specified. Cells were harvested at the indicated time points for protein extraction and Western blot analysis.

### Western blot

Protein extraction from pancreatic cancer tissue samples and cultured cells was performed using RIPA buffer, followed by incubation on ice for 30 minutes. After centrifugation at 12,000 rpm for 20 minutes at 4°C, the supernatant was collected for protein analysis. Protein concentrations were determined using the BCA assay kit (Thermo Fisher). Equal amounts of protein were loaded onto a 10% SDS-PAGE gel and transferred to a PVDF membrane (Millipore). The membrane was blocked with 5% non-fat milk in TBST for 1 hour at room temperature, followed by overnight incubation at 4°C with primary antibodies: TRIM9 (ProteinTech, #10786-1-AP), actin (ProteinTech, #66009-1-Ig), anti-Flag (Cell Signaling Technology, #14793), anti-Myc (Cell Signaling Technology, #2276), and anti-HA (Cell Signaling Technology, #3724). After washing with TBST, the membrane was incubated with HRP-conjugated secondary antibodies for 1 hour at room temperature. Protein bands were visualized using an enhanced chemiluminescence (ECL) detection system (Bio-Rad).

### RT-qPCR

Total RNA was extracted from cultured cells or tissue samples using TRIzol reagent (Invitroge), and RNA quality was confirmed using a NanoDrop spectrophotometer. High-quality RNA was reverse-transcribed into cDNA using the PrimeScript RT reagent kit (Takara). Quantitative PCR (qPCR) was performed using SYBR Green PCR Master Mix (Applied Biosystem) with TRIM9-specific primers and actin as the internal control. The qPCR reaction was conducted on a real-time PCR system under the following conditions: initial denaturation at 95°C for 10 minutes, followed by 40 cycles of 95°C for 15 seconds and 60°C for 60 seconds. The relative expression levels of TRIM9 were calculated using the 2^(-ΔΔCt) method and normalized to actin. All experiments were performed in triplicate to ensure reproducibility.

### Co-immunoprecipitation methodology

Cells were lysed using IP lysis buffer (Thermo Fisher) supplemented with protease and phosphatase inhibitors. The lysates were centrifuged at 12,000 rpm for 15 minutes at 4°C to remove debris. Supernatants were incubated with 2-5 µg of target-specific primary antibody or IgG control overnight at 4°C with rotation, followed by incubation with Protein A/G magnetic beads (Thermo Fisher) for 2 hours at 4°C. Beads were washed three times with lysis buffer, and bound proteins were eluted using SDS loading buffer by heating at 95°C for 5 minutes. The eluted proteins were analyzed by Western blot to detect interactions.

### Subcutaneous xenograft tumor model

To evaluate tumor growth *in vivo*, six-week-old male Balb/c nude mice (GemPharmatech Co., Ltd) were selected for implantation. MIAPaCa-2 pancreatic cancer cells, after transfection with the designated constructs following standard procedures, were resuspended in 100 µL of DMEM mixed with Matrigel at a concentration of 2 × 10^6^ viable cells. The cell suspension was injected subcutaneously into the right flank under sterile conditions.

Tumor size was recorded every three days by measuring length and width with a digital caliper. The tumor volume was estimated using the formula: 0.5 × Length × Width². At the study’s conclusion, mice were euthanized in accordance with ethical protocols, and tumors were harvested, weighed, and preserved for subsequent histopathological and molecular analyses.

### Statistical methods

Gene expression across different groups was compared using the rank-sum test. Survival analysis was conducted using the Kaplan-Meier (KM) method with the log-rank test. Correlations between continuous variables were analyzed using Pearson’s method. All analyses were performed using R software (version 4.0.5). A p-value of <0.05 was considered statistically significant. “*” means “p<0.05”, “**” means “p<0.01”, “*” means “p<0.001”.

## Results

### Single-cell sequencing landscape of ubiquitination-related genes in pancreatic cancer

**Figure 1:**
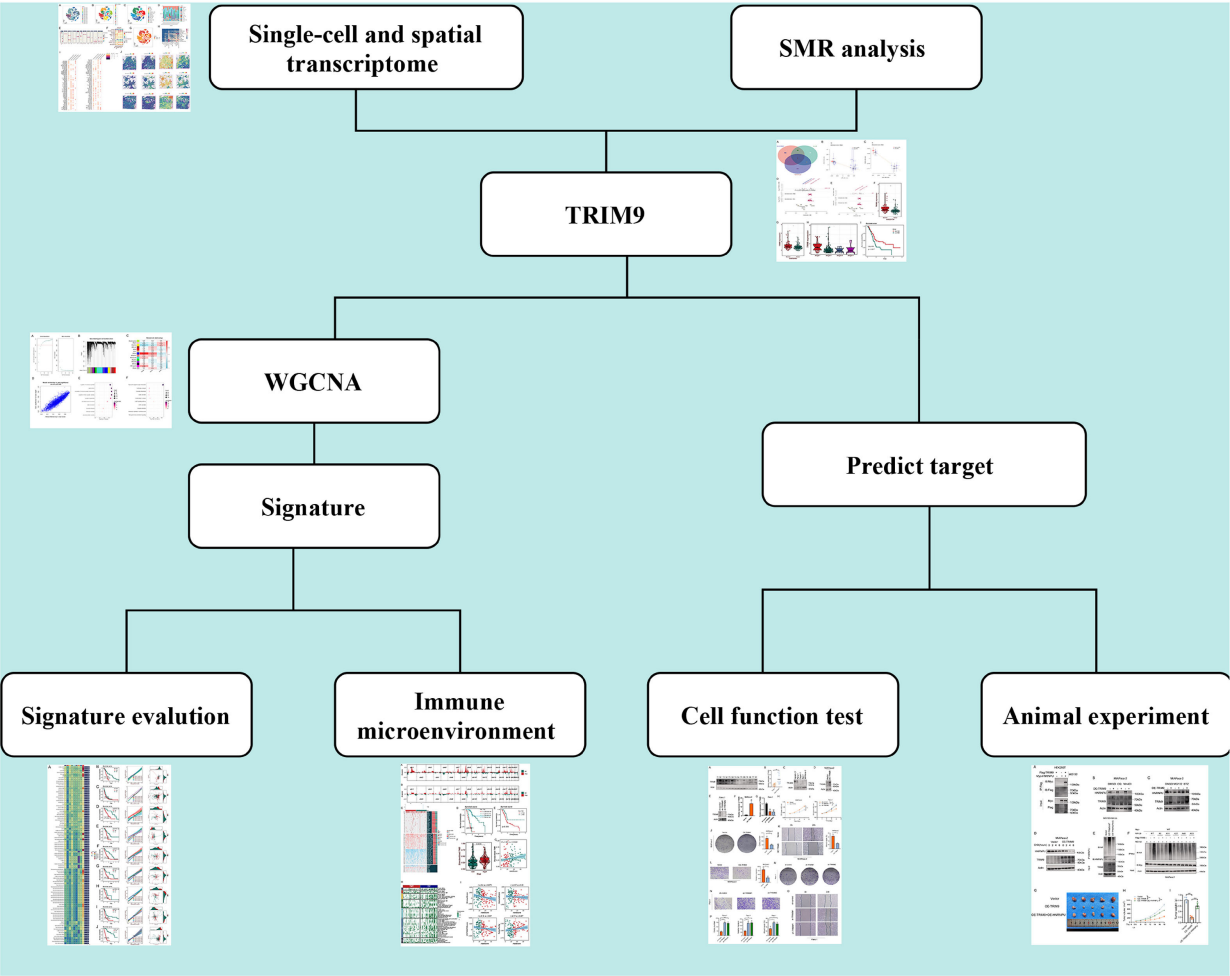
Overview of the study design. The schematic representation illustrates the workflow, including data preprocessing, single-cell analysis, machine learning-based prognostic modeling, and functional validation experiments.

**Figure 2:**
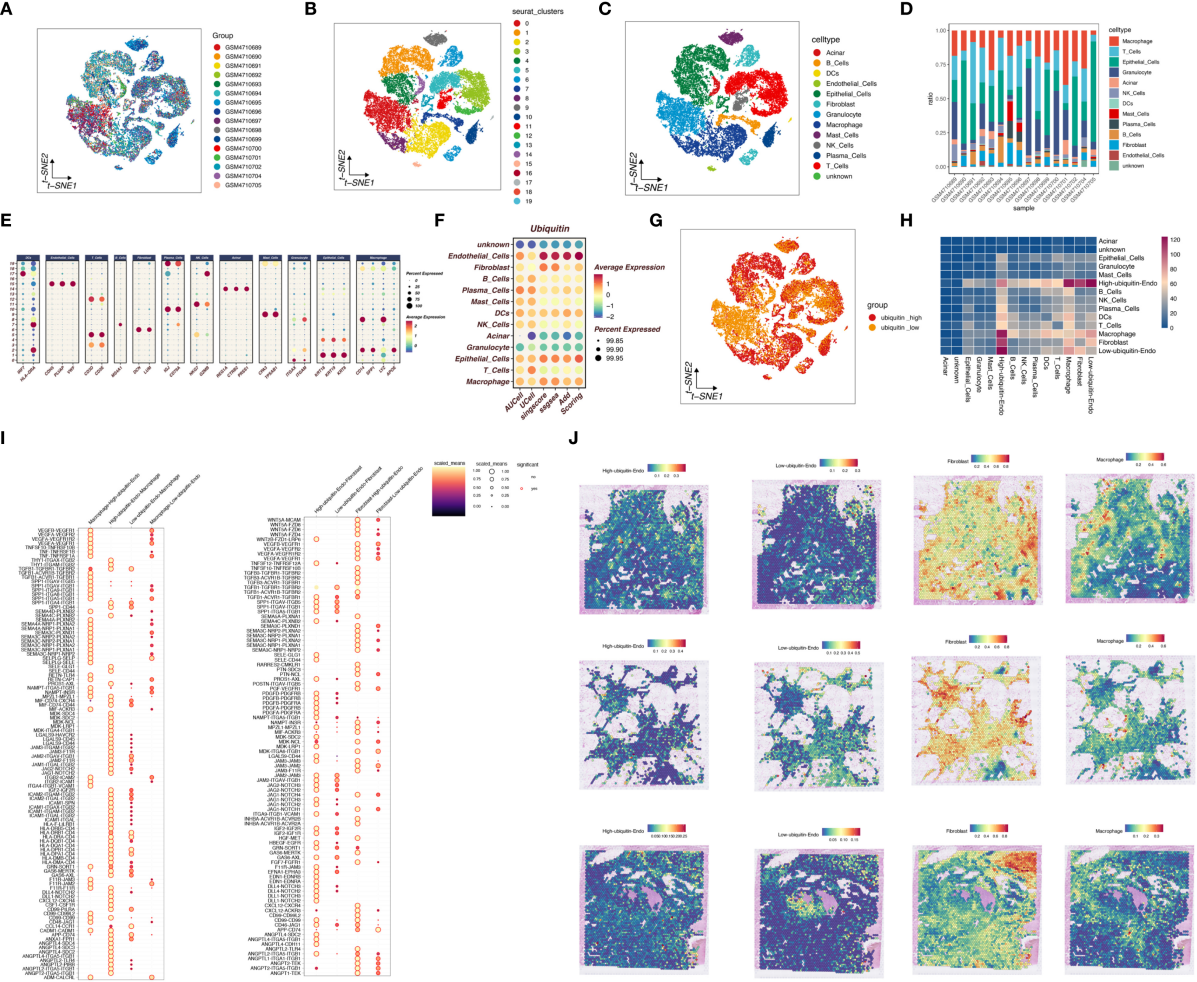
Single-cell sequencing analysis of ubiquitination-related genes in pancreatic cancer. **(A)** Quality control and sample integration, showing retained tumor samples with minimal batch effects. **(B, C)** Clustering of tumor samples into distinct cell types based on marker gene expression. **(D, E)** Proportions of different cell types, highlighting the enrichment of epithelial cells, T cells, and macrophages. **(F, G)** Ubiquitination phenotype scores for each cell type, revealing high ubiquitination in endothelial cells. **(H)** Cell-cell communication analysis showing interactions between highly ubiquitinated endothelial cells, macrophages, and fibroblasts. **(I)** Ligand-receptor interactions identified in High_ubiquitin-Endo cells. **(J)** Spatial distribution analysis confirming proximity of High_ubiquitin-Endo cells to fibroblasts and macrophages.

The workflow of this study is shown in **Figure 1**. We explored the ubiquitination phenotype at the single-cell level in pancreatic cancer. As shown in **Figure 2A**, after quality control and sample integration, 16 tumor samples were retained, with no significant batch effects observed between the samples. As shown in **Figures 2B, C**, the tumor samples were clustered into 20 clusters, which were annotated as various cell types. Based on the expression of cell-type-specific marker genes, 12 cell types were annotated, with a higher proportion of epithelial cells, T cells, and macrophages (**Figures 2D, E**). Subsequently, as shown in **Figures 2F, G**, the ubiquitination phenotype score was quantified for each cell, revealing that endothelial cells had a higher ubiquitination score. Based on the average ubiquitination score of endothelial cells derived from multiple methods, we classified them into high-ubiquitination endothelial cells (High_ubiquitin-Endo) and low-ubiquitination endothelial cells (Low_ubiquitin-Endo). To explore the interactions between endothelial cells and other cell types, we conducted a cell-cell communication analysis. As shown in **Figure 2H**, compared to Low_ubiquitin-Endo cells, High_ubiquitin-Endo cells were more closely associated with macrophages and fibroblasts. As shown in **Figure 2I**, WNT2B, FZD1, and LRP6, as well as DLL1, NOTCH3, and NOTCH2, might play important roles in the communication between High_ubiquitin-Endo cells and fibroblasts, while THY1, ITGAX, ITGAM, ITGB2, SELE, GLG1, and CD44 might be involved in the communication between High_ubiquitin-Endo cells and macrophages. To further investigate the distribution of cells at the transcriptional level, we performed deconvolution analysis of the blank-control and single-cell data to obtain the cell types in the blank-control samples. As shown in **Figure 2J**, in the samples GSM7498811, GSM7498812, and GSM7498813, High_ubiquitin-Endo cells were primarily distributed around fibroblasts and macrophages, suggesting a potential close interaction between these cell types.

### Mendelian randomization SMR analysis to identify ubiquitination-related genes closely associated with pancreatic cancer

**Figure 3:**
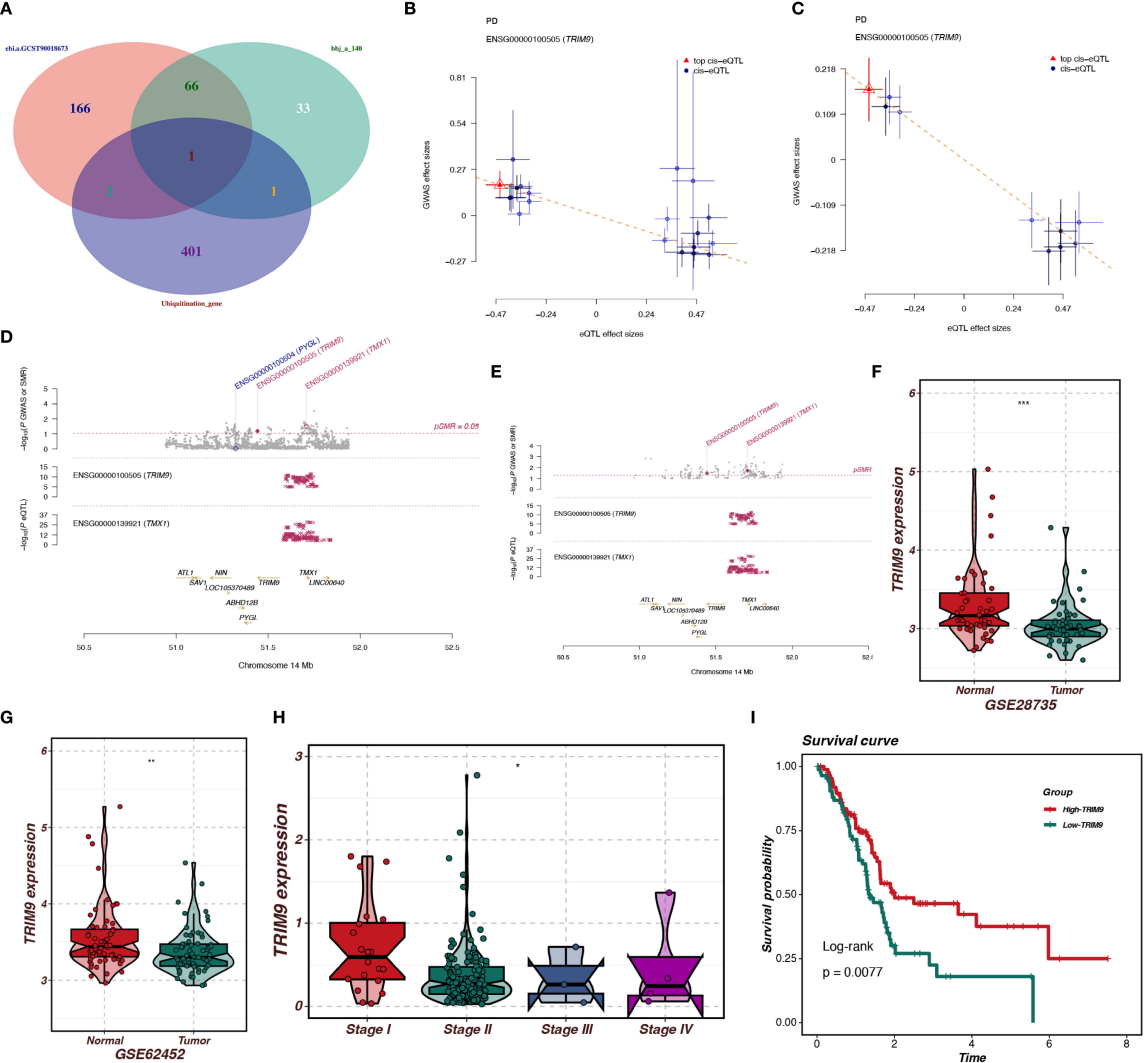
SMR analysis identifies ubiquitination-related genes associated with pancreatic cancer. **(A)** Intersection of ubiquitination-related and pancreatic cancer-associated genes, identifying TRIM9. **(B-E)** TRIM9 is associated with TMX1 and PYGL, indicating a protective role. **(F, G)** Differential expression analysis shows TRIM9 downregulation in pancreatic cancer tissues. **(H, I)** TRIM9 expression decreases with tumor grade; Kaplan-Meier survival analysis indicates that higher TRIM9 expression correlates with better prognosis.

In this study, we employed SMR analysis to identify genes related to ubiquitination and closely associated with pancreatic cancer onset. Using the bbj_a_140 dataset and the ebi-a-GCST90018673 dataset, we performed SMR analysis with the criteria p_SMR < 0.05 and p_HEIDI > 0.05, which identified 101 and 235 pancreatic cancer-associated genes, respectively. As shown in **Figure 3A**, by intersecting the ubiquitination-related genes with pancreatic cancer-associated genes, we identified a gene, TRIM9. As shown in **Figures 3B-E**, TRIM9 was found to be a protective factor for pancreatic cancer in both the bbj_a_140 and ebi-a-GCST90018673 datasets, and it exhibited colocalization with genes such as TMX1 and PYGL.

To further explore the role of TRIM9 in pancreatic cancer, we conducted expression and prognosis analyses. As shown in **Figures 3F, G**, in the GSE287735 and GSE62452 datasets, TRIM9 was found to be downregulated in pancreatic cancer tissues compared to normal tissues (p<0.001, p<0.01). As shown in **Figures 3H, I**, in the TCGA cohort, we observed differential expression of TRIM9 across tumor stages, with TRIM9 expression decreasing as tumor grade increased. Kaplan-Meier (KM) analysis indicated that patients with higher TRIM9 expression had a better prognosis (p<0.05).

### WGCNA analysis

**Figure 4:**
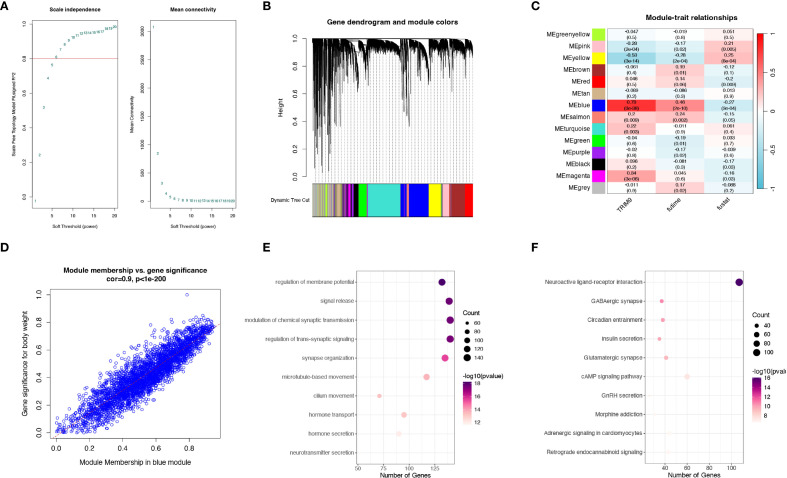
WGCNA analysis of TRIM9 co-expressed genes. **(A)** Optimal soft threshold selection for WGCNA. **(B, C)** Gene clustering and identification of the module most correlated with TRIM9 and prognosis. **(D)** Gene significance analysis in the key module. **(E, F)** Functional enrichment analysis highlighting pathways linked to TRIM9 co-expressed genes.

To identify the co-expressed genes of TRIM9, we performed WGCNA analysis in the TCGA cohort. As shown in **Figure 4A**, the optimal soft threshold was calculated to be 7, and the data followed a power-law distribution. As shown in **Figures 4B, C**, genes were clustered into 13 non-gray modules, with the blue module showing the highest correlation with TRIM9 (cor = 0.79, p < 0.05), and this module was also significantly associated with prognosis (p < 0.05). We further investigated the gene correlations within the green module. As shown in **Figure 4D**, the module membership in the green module was strongly positively correlated with the gene significance for body weight (cor = 0.9, p < 0.05). As shown in **Figures 4E, F**, the genes in the blue module were primarily involved in intercellular signaling processes.

### Machine learning approaches for constructing and validating prognostic models

**Figure 5:**
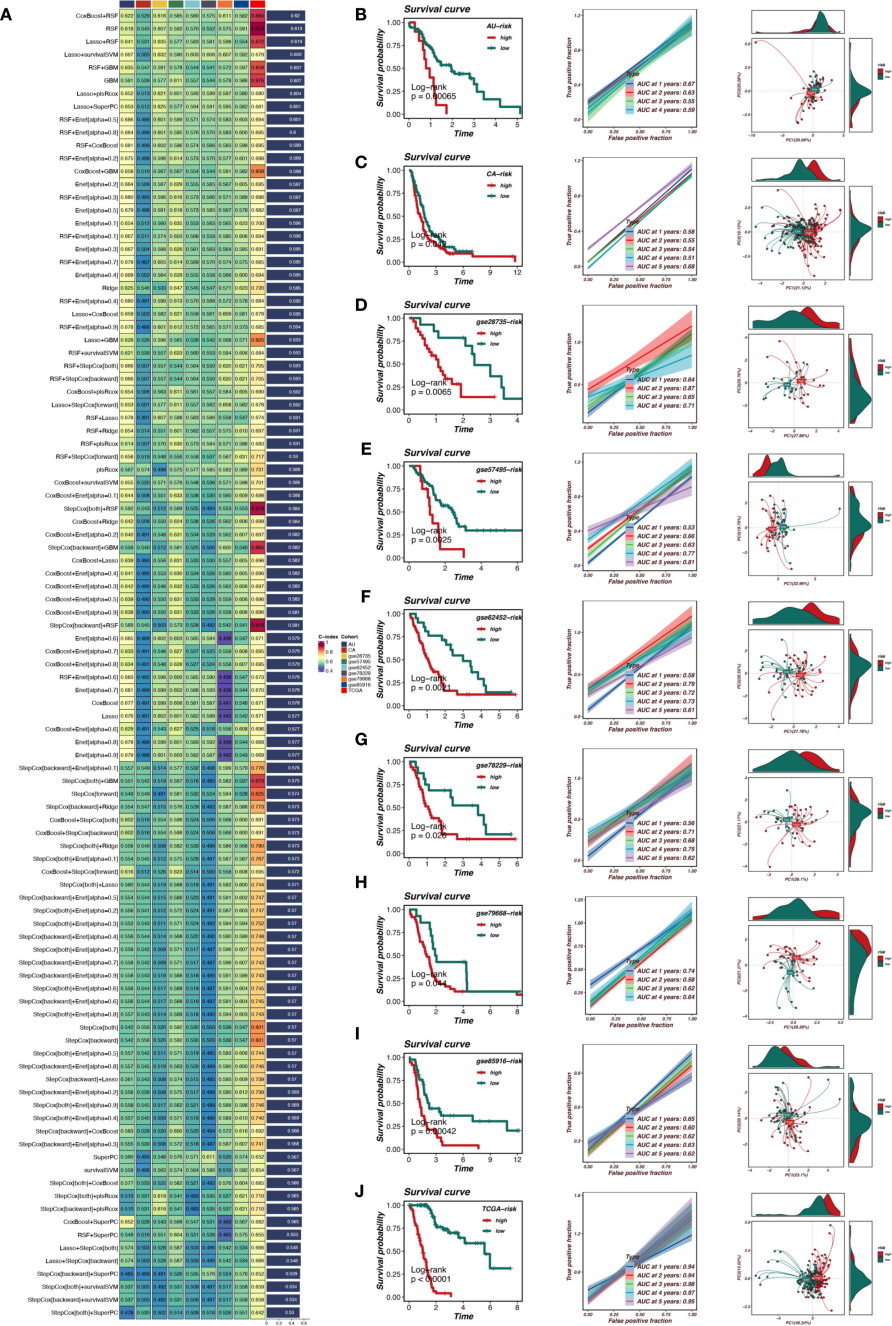
Machine learning-based prognostic model for pancreatic cancer. **(A)** Selection of the optimal machine learning model using CoxBoost+RSF. **(B-J)** Validation of the prognostic model across multiple datasets, demonstrating significant stratification of high-risk and low-risk patients with strong predictive accuracy.

In this study, we constructed a prognostic model based on TRIM9 co-expressed genes. We selected genes from the blue module that were closely associated with TRIM9 and present across all datasets. First, univariate Cox analysis was performed with a threshold of P<0.05 to identify genes associated with prognosis. Subsequently, a prognostic model was constructed using a combination of multiple machine learning methods in the TCGA cohort and validated in other cohorts. As shown in **Figure 5A**, the optimal machine learning combination was CoxBoost+RSF, which resulted in the highest average C-index across all cohorts. This model included 7 genes: TSPAN6, TSC1, RNF167, PBXIP1, LRRC49, KATNAL2, and IGF2BP2. Patients were stratified into high-risk (risk_high) and low-risk (risk_low) groups based on the median model score. As shown in **Figures 5B-J**, patients in the risk_high group had worse prognosis, and overall, the model demonstrated high accuracy in prognostic prediction. There was a significant difference in prognosis between the risk_high and risk_low groups across the TCGA, AU, CA, GSE28735, GSE57495, GSE62452, GSE78229, GSE79668, and GSE85916 cohorts.

### Mutation and immune-related analysis

**Figure 6:**
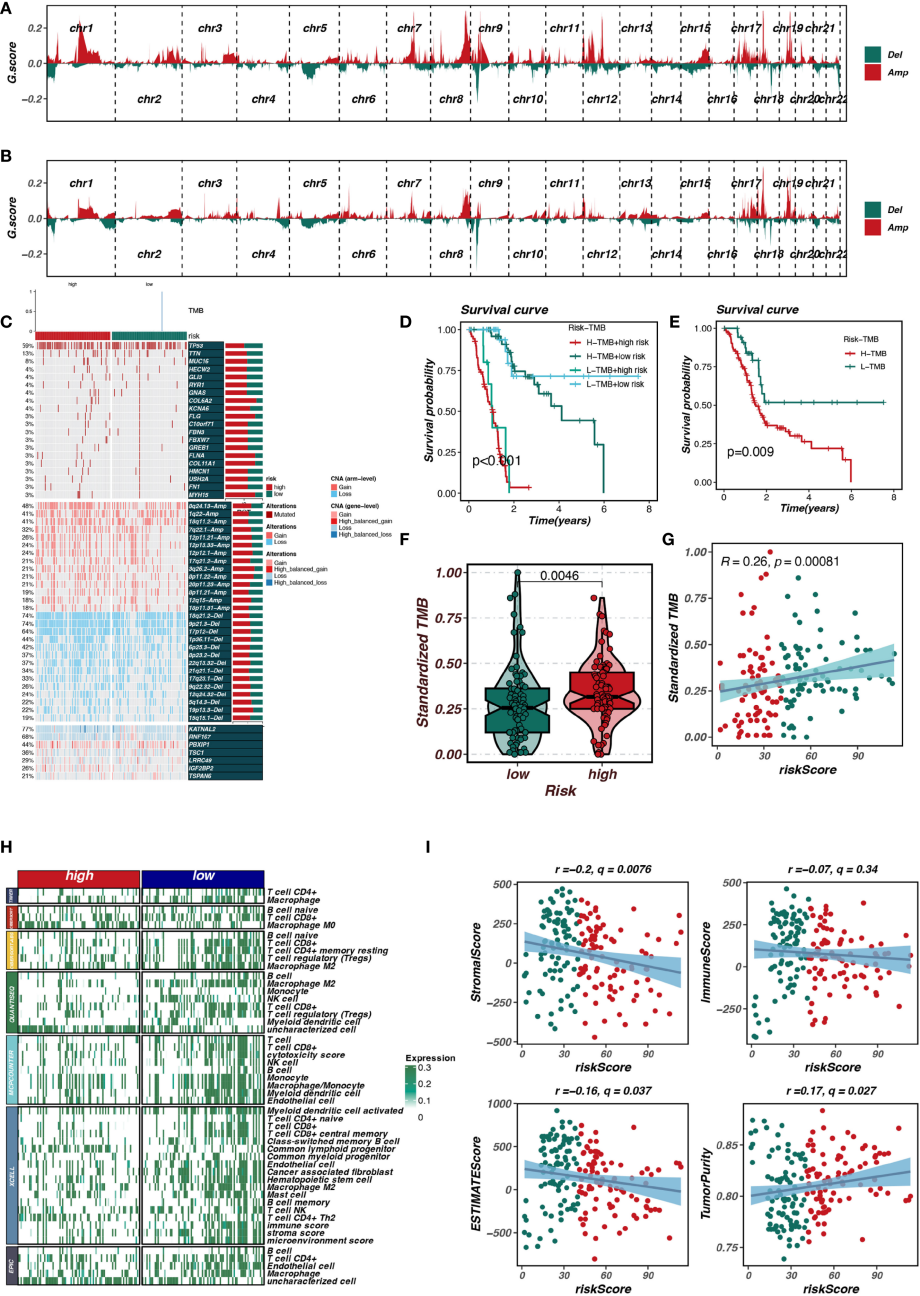
Mutation and immune-related analysis of risk groups. **(A, B)** Comparison of gene mutations in high-risk and low-risk groups. **(C)** Higher mutation frequencies of TP53 and TTN in the high-risk group. **(D, E)** Association of high tumor mutational burden (TMB) with poor prognosis. **(F, G)** Correlation between risk score and TMB. **(H, I)** Differences in immune cell infiltration and tumor purity between risk groups.

This study further explored the differences in copy number variation, gene mutations, and immune cell infiltration between the risk_high and risk_low groups. As shown in **Figures 6A, B**, the gene mutation status in the risk_high and risk_low groups was compared, with the risk_high group showing a trend of more gene deletions and new mutations. As shown in **Figure 6C**, the risk_high group had a higher frequency of mutations in genes such as TP53 and TTN. **Figures 6D, E** demonstrate that pancreatic cancer patients with higher TMB have worse prognosis compared to those with lower TMB, and patients with high TMB in the risk_high group had the worst prognosis. As shown in **Figures 6F, G**, patients in the risk_high group had higher TMB, and the riskscore was positively correlated with TMB (R = 0.26, p < 0.05). **Figures 6H, I** show that patients in the risk_high group had lower immune cell infiltration, including T cells and B cells, and the riskscore was positively correlated with tumor purity and negatively correlated with stromal and immune scores (p < 0.05).

Additionally, we analyzed the relationships between TRIM9, model genes, and the riskScore. As shown in **Supplementary Figure S1**, TRIM9 was significantly positively correlated with genes such as TSC1, RNF167, PBXIP1, LRRC49, and KATNAL2 (p < 0.05), while it was significantly negatively correlated with TSPAN6 and IGF2BP2.

### Low expression of TRIM9 in pancreatic cancer and its tumor-suppressive role

**Figure 7:**
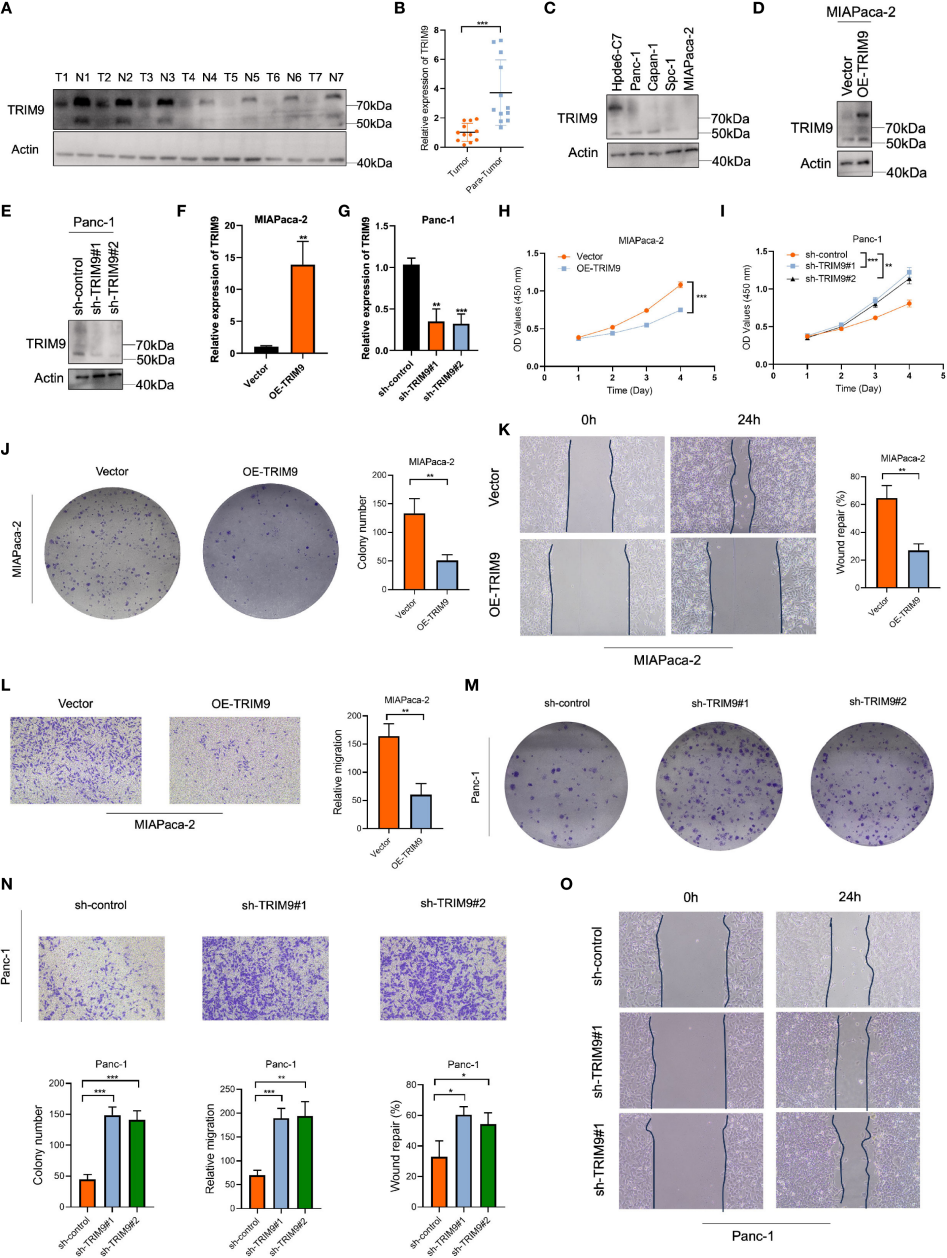
TRIM9 expression and functional validation in pancreatic cancer. **(A, B)** Western blot and PCR confirm lower TRIM9 expression in pancreatic cancer tissues. **(C)** TRIM9 expression levels in pancreatic cancer cell lines. **(D-G)** Validation of overexpression and knockdown efficiency in MIAPaca-2 and Panc-1 cells. **(H, I)** CCK-8 assay showing TRIM9 overexpression reduces cell viability, while knockdown enhances viability. **(J-O)** Colony formation, wound healing, and Transwell assays demonstrate that TRIM9 suppresses proliferation and migration. *p < 0.05, **p< 0.01, ***p< 0.001.

To verify the role of TRIM9 in pancreatic cancer, we first performed Western blot (WB) and PCR analyses on clinical samples. Both WB and PCR results showed that TRIM9 expression was significantly downregulated in pancreatic cancer tissues compared to adjacent normal tissues (**Figures 7A, B**). Similarly, in pancreatic cancer cell lines, TRIM9 expression was also reduced compared to normal pancreatic cell lines, with the lowest expression observed in the MIAPaca-2 cell line and relatively higher expression in the Panc-1 cell line (**Figure 7C**). Based on these findings, we overexpressed TRIM9 in MIAPaca-2 and knocked down TRIM9 in Panc-1, with overexpression and knockdown efficiency validated by WB and PCR (**Figures 7D-G**). Subsequently, a series of cell assays were conducted. First, the CCK-8 assay showed that overexpression of TRIM9 in MIAPaca-2 cells significantly reduced cell viability (**Figure 7H**), while knocking down TRIM9 in Panc-1 cells significantly enhanced cell viability (**Figure 7I**). Similarly, overexpression of TRIM9 in MIAPaca-2 cells resulted in significantly decreased proliferation and migration, as shown by colony formation, wound healing, and Transwell migration assays (**Figures 7J-L**). Conversely, knockdown of TRIM9 in Panc-1 cells significantly enhanced cell proliferation and migration (**Figures 7M-P**).

### TRIM9 reduces HNRNPU protein stability through K11-linked ubiquitination

**Figure 8:**
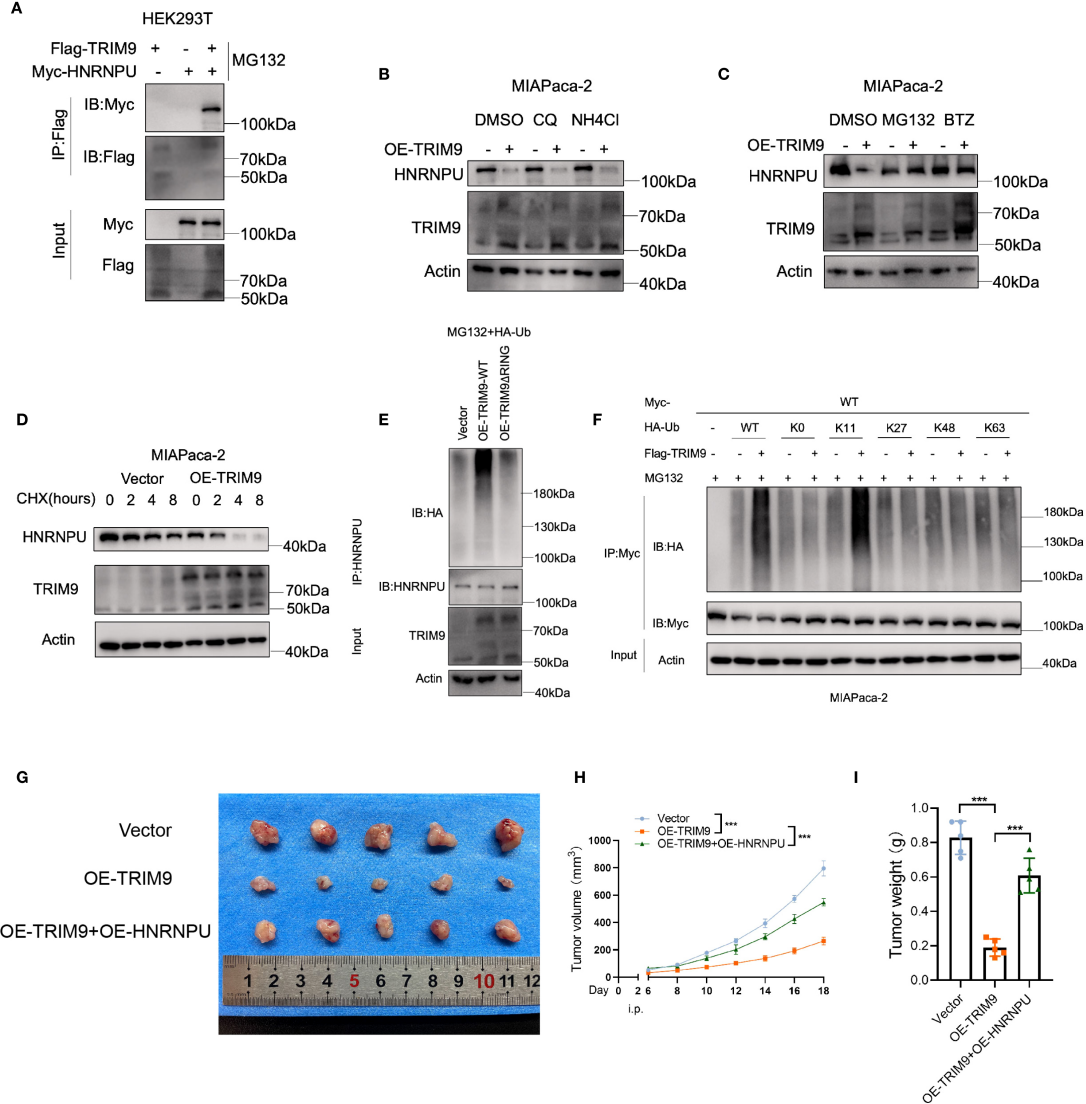
TRIM9-mediated ubiquitination of HNRNPU. **(A)** Co-immunoprecipitation confirms TRIM9 interaction with HNRNPU. **(B)** Ubiquitination assays reveal TRIM9 promotes K11-linked ubiquitination of HNRNPU, leading to its degradation. **(C)** Functional validation of the TRIM9-HNRNPU axis in pancreatic cancer progression. **(D)** Overexpression of TRIM9 significantly decreased the stability of HNRNPU protein. **(E, F)** TRIM9 modifies HNRNPU through K11-linked ubiquitination. **(G–I)** The role of TRIM9 and HNRNPU in vivo experiments. ***p< 0.001.

In HEK293T cells, we conducted co-immunoprecipitation and found that Flag-tagged TRIM9 protein can co-precipitate with Myc-tagged HNRNPU (**Figure 8A**), indicating an interaction between the two proteins. We then investigated the relationship between TRIM9 and the stability of HNRNPU protein. In the MIAPaca-2 pancreatic cancer cell line, overexpression of TRIM9 led to a reduction in the protein level of HNRNPU. This effect could not be reversed by lysosomal inhibitors CQ and NH4Cl (**Figure 8B**), but could be reversed by proteasomal inhibitors MG132 and BTZ (**Figure 8C**), suggesting that TRIM9 mediates the degradation of HNRNPU through the proteasomal pathway. Further, a cycloheximide (CHX) chase experiment revealed that overexpression of TRIM9 significantly decreased the stability of HNRNPU protein (**Figure 8D**).

To investigate the underlying mechanism, we assessed the ubiquitination level of HNRNPU by co-immunoprecipitation, revealing that the presence of the RING domain in TRIM9 is essential for its ubiquitin modification of HNRNPU (**Figure 8E**). Moreover, by transfecting various mutant HA-Ub plasmids, we identified that TRIM9 modifies HNRNPU through K11-linked ubiquitination (**Figure 8F**).

Further *in vivo* experiments demonstrated that overexpression of TRIM9 reduced the tumor volume and weight in nude mice, while co-overexpression of HNRNPU rescued this effect (**Figures 8G-I**).

## Discussion

Pancreatic cancer (PC) remains one of the deadliest malignancies, with an overall survival rate of less than 10% and limited treatment options available to patients (24). Despite substantial progress in the molecular characterization of PC, the precise molecular mechanisms that underlie tumor progression and resistance to therapy remain incompletely understood (25). A growing body of evidence has implicated post-translational modifications, especially ubiquitination, as central regulatory mechanisms in cancer biology. Ubiquitination, by modulating protein stability, activity, and subcellular localization, controls a wide array of cellular processes, including cell cycle progression, apoptosis, DNA repair, and protein degradation. Immunotherapy plays a significant role in cancer treatment (26). In this study, we identified TRIM9 as a critical player in regulating the stability of HNRNPU, a key RNA-binding protein involved in tumor progression, via K11-linked ubiquitination. Our findings suggest that TRIM9-mediated ubiquitination represents a novel mechanism for regulating protein stability in pancreatic cancer and may offer new therapeutic avenues for targeting ubiquitination-related pathways.

The first step in our research involved the systematic analysis of ubiquitination-related genes, which revealed a broad landscape of genes potentially involved in pancreatic cancer. Among these, TRIM9 stood out due to its functional relevance in various cellular processes, including protein degradation, immune response regulation, and stress signaling. While previous studies have investigated the role of TRIM9 in cellular homeostasis, its role in cancer, particularly in pancreatic cancer, had not been well characterized. By integrating transcriptomic data and gene co-expression networks, we uncovered that TRIM9 is significantly associated with various cancer-related pathways, highlighting its potential as a key modulator of tumorigenesis.

TRIM9 is a member of the TRIM family, which is known for its involvement in ubiquitination and the regulation of protein turnover (27). However, the functional specificity of individual TRIM family members in different cancer types is still not fully understood. Our study contributes to this gap by providing evidence that TRIM9 exerts its effects on pancreatic cancer through its interaction with HNRNPU. HNRNPU is an RNA-binding protein that regulates alternative splicing, RNA stability, and transcriptional control, and has been shown to promote tumor cell proliferation and survival in various cancers, including pancreatic cancer (28). However, the regulation of HNRNPU stability, particularly through post-translational modifications, remains poorly understood. Our data suggest that TRIM9 can target HNRNPU for degradation via K11-linked ubiquitination, leading to a decrease in HNRNPU protein levels. This represents a novel regulatory mechanism by which TRIM9 modulates the stability of a key oncogenic protein in pancreatic cancer.

Furthermore, the specific involvement of K11-linked ubiquitination in the regulation of HNRNPU stability is of particular interest. HNRNPU has been proven to be highly expressed in pancreatic cancer and is associated with poor prognosis (29–31).While K48-linked ubiquitination is the canonical signal for proteasomal degradation, other types of ubiquitination, such as K11-linked, have recently emerged as important regulators of protein stability and function. K11-linked ubiquitination is known to play a role in the regulation of the cell cycle and the maintenance of genomic stability, which are both critical aspects of cancer biology. The identification of K11-linked ubiquitination as a key mechanism for regulating HNRNPU provides new insights into the complexity of the ubiquitination machinery and its role in pancreatic cancer. Future studies will be required to further elucidate the molecular pathways that link K11-linked ubiquitination to the regulation of HNRNPU, as well as the potential crosstalk between different types of ubiquitin chains in this context.

Our findings also suggest that targeting TRIM9 or its associated ubiquitination machinery could offer novel therapeutic opportunities for pancreatic cancer. Currently, there are limited therapeutic strategies that directly target the ubiquitin-proteasome system (UPS), despite its central role in regulating various oncogenic pathways. Proteasome inhibitors such as bortezomib have shown promise in the treatment of multiple myeloma and other cancers; however, their effectiveness in solid tumors like pancreatic cancer is still being explored. Given the essential role of TRIM9 in modulating HNRNPU stability and tumor progression, targeting TRIM9-mediated ubiquitination could provide a more specific approach to modulate protein degradation pathways in pancreatic cancer. This could potentially improve treatment outcomes and overcome some of the resistance mechanisms associated with conventional therapies.

Moreover, the integration of single-cell RNA sequencing and spatial transcriptomics in our study provided valuable insights into the cellular heterogeneity and spatial distribution of TRIM9 and HNRNPU within the tumor microenvironment. The ability to analyze these molecules at a single-cell resolution enables a more detailed understanding of their roles in distinct cell populations, such as cancer stem cells, immune cells, and stromal cells, all of which contribute to pancreatic cancer progression. Future studies incorporating single-cell proteomics and spatially resolved proteomics will further enhance our understanding of the dynamic interactions between TRIM9, HNRNPU, and other tumor-associated factors.

In conclusion, our study identifies TRIM9 as a key regulator of HNRNPU stability through K11-linked ubiquitination in pancreatic cancer. This novel mechanism highlights the complexity of ubiquitination in regulating protein turnover and tumor progression. By further investigating the role of TRIM9 and its associated pathways in pancreatic cancer, we may uncover new therapeutic strategies to target ubiquitination-related processes and improve patient outcomes.

## Data Availability

The original contributions presented in the study are included in the article/**Supplementary Material**. Further inquiries can be directed to the corresponding author.

## Supplementary material

Supplementary Figure 1 Associations between TRIM9 and model genes and risk scores.

Supplementary Table 1 The PCR primer.
