# Supplementary figures and images for: Single-cell and multi-omics analysis identifies TRIM9 as a key ubiquitination regulator in pancreatic cancer
Source: Front Immunol. 2025 Sep 19;16:1631708. doi: 10.3389/fimmu.2025.1631708 (PMC12491318; doi:10.3389/fimmu.2025.1631708)

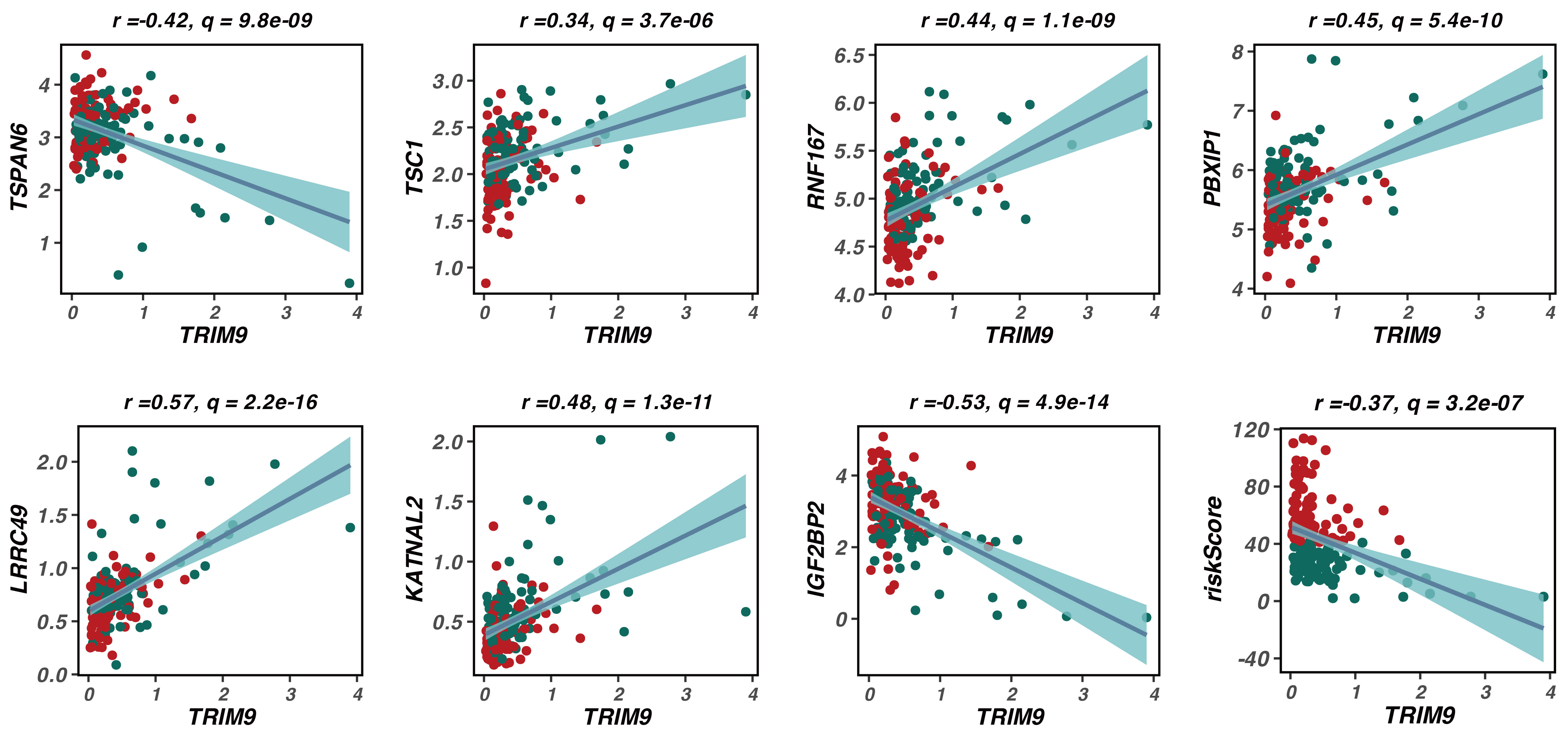

Supplement: Supplementary Figure 1 — Associations between TRIM9 and model genes and risk scores. [file Image1.jpeg]

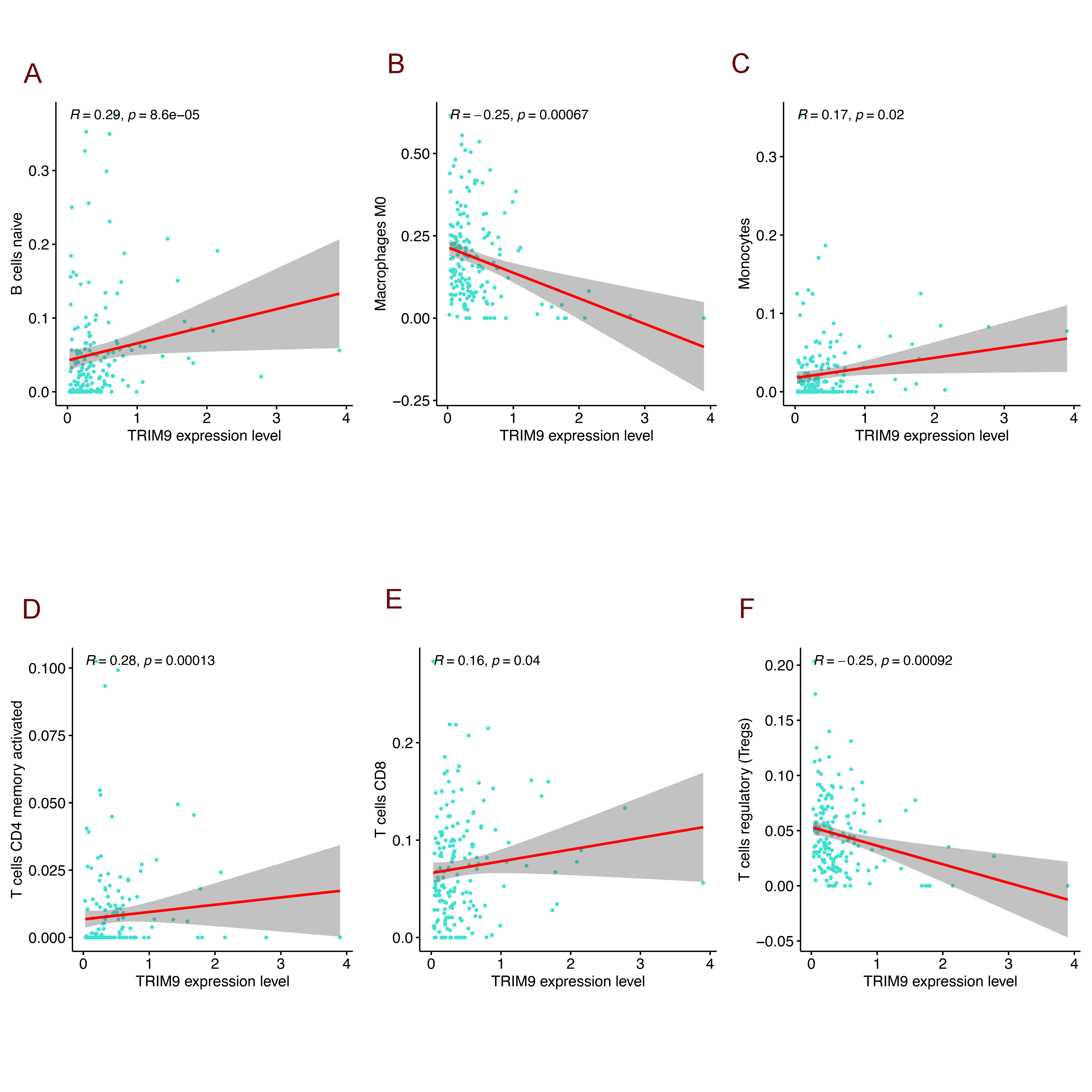

Supplement: Supplementary file 3 [file Image2.tif]

Figure 7A

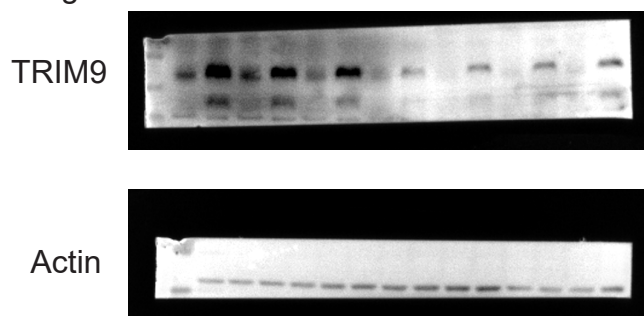

Figure 7C

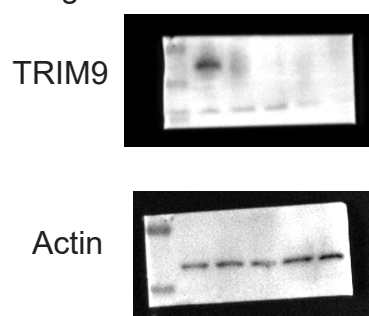

Figure 7D

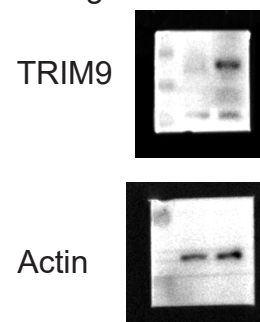

Figure 7E

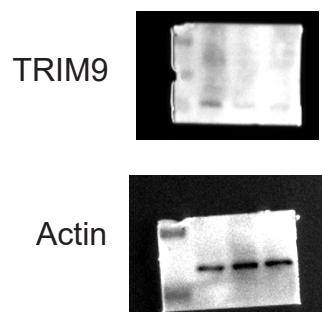

Figure 8A

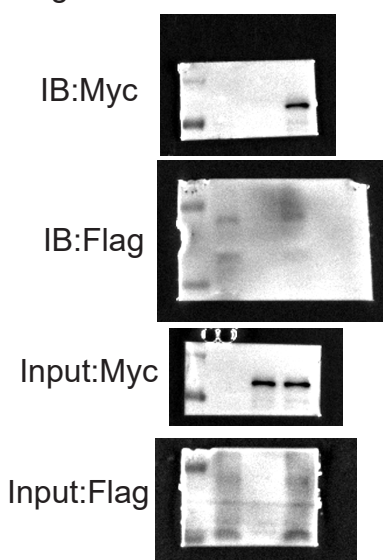

Figure 8B

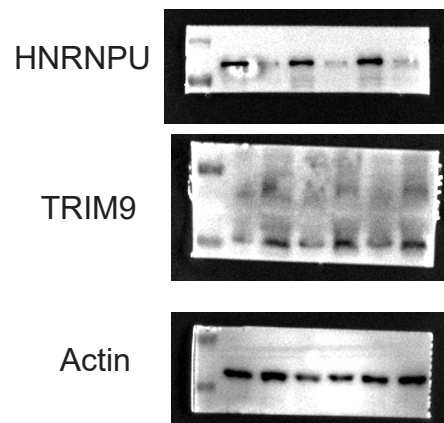

Figure 8E

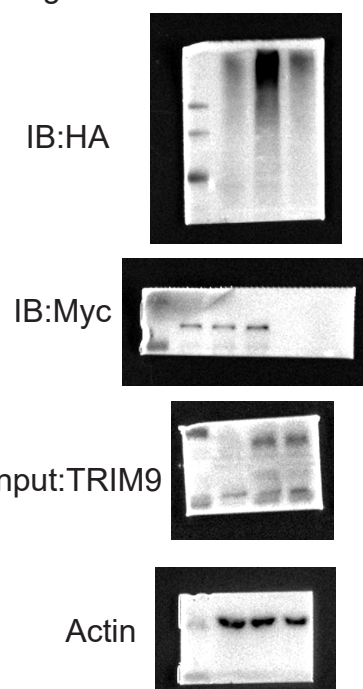

Figure 8C

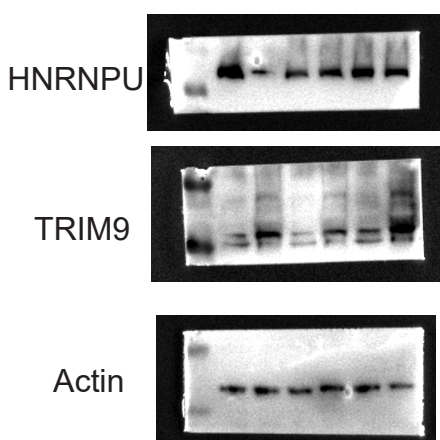

Figure 8D

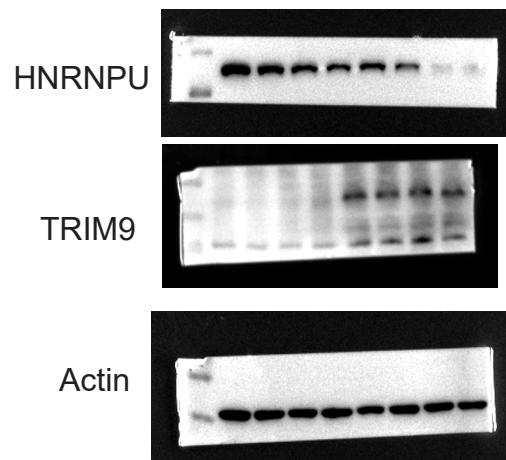

Figure 8F

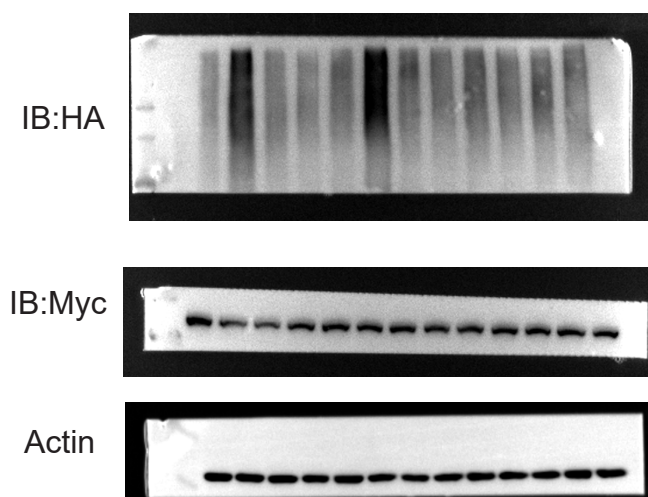

Supplement: Supplementary file 4 [file DataSheet2.pdf]
