# Supplementary material for: Single-cell and multi-omics analysis identifies TRIM9 as a key ubiquitination regulator in pancreatic cancer
Source: Front Immunol. 2025 Sep 19;16:1631708. doi: 10.3389/fimmu.2025.1631708 (PMC12491318; doi:10.3389/fimmu.2025.1631708)
Supplement: Supplementary Table 1 — The PCR primer. [file Table1.docx]

Supplementary Table 1. PCR primers.

TRIM9:

Forward: AAGCTTCCTCTCCAGTCCCA

Reverse: CCTCAGACGTTTGGAGGACC

ACTB：

Forward: TCACCATGGATGATGATATCGC

Reverse: ATAGGAATCCTTCTGACCCATGC
