# Supplementary material for: Single-cell and multi-omics analysis identifies TRIM9 as a key ubiquitination regulator in pancreatic cancer
Source: Front Immunol. 2025 Sep 19;16:1631708. doi: 10.3389/fimmu.2025.1631708 (PMC12491318; doi:10.3389/fimmu.2025.1631708)
Supplement: Supplementary file 5 [file DataSheet4.pdf]

伦理审查批件

批件号： 2025-LP-034

**项目名称**	泛素化相关基因TRIM9通过调控HNRNPU在胰腺癌中抑制肿瘤进展		
**受理号**	2025-KY-026		
**项目负责人**	陈亮	**承担科室**	肝胆胰外科
**合作单位**	无		
**审查类别**	初始审查	**审查方式**	简易程序审查
**审查日期**	2025.01.15	**审查地点**	各自办公室
**审查文件清单**	1. 科研课题伦理审查申报表<br>2. 研究方案<br>3. 技术路线<br>4. 知情同意书		
**审查决定**	批准		
**主任/副主任委员签字**	[illegible]		
**签发日期**	2025 年 01 月 17 日		
**伦理委员会**	嘉兴市第一医院医学伦理委员会（盖章）		
**批件有效期**	自本伦理委员会初始审查批准之日起一年内，本临床研究应在本院启动。逾期未启动的，本批件自行废止。		
**年度/定期跟踪审查频率**	审查频率为该研究批准之日起每 12 月一次，首次年度/定期跟踪审查请于 2026年01月16日前1个月递交研究进展报告。<br>伦理委员会有根据实际进展情况改变跟踪审查频率的权利。		
**声明**	本伦理委员会的职责、人员组成、操作程序及记录遵循《涉及人的生物医学研究伦理审查办法》、《涉及人的健康相关研究国际伦理准则》、《赫尔辛基宣言》等伦理指南和相关法律法规。		

注意事项：

1. 请遵循我国相关法律、法规和规章中的伦理原则。
2. 请遵循经本伦理委员会批准的临床研究方案、知情同意书、招募材料等开展本研究，保护研究参与者的健康与权利。对研究方案、知情同意书和招募材料等的任何修改，均须得到本伦理委员会审查同意后方可实施。
3. 研究期间发生的SAE及时评估后递交本伦理委员会。
4. 根据报告情况，本伦理委员会有权对其评估做出新的决定。
5. 自今日起，无论研究开始与否，请在跟踪审查日到期前1个月提交研究进展报告。
6. 研究纳入了不符合纳入标准或符合排除标准的研究参与者，符合中止研究规定而未让研究参与者退出研究，给予错误治疗或剂量，给予方案禁止的合并用药等没有遵从方案开展研究的情况；或可能对研究参与者的权益或健康以及研究的科学性造成不良影响等情况，请研究者提交违背方案报告。
7. 研究者暂停或提前终止临床研究，请及时提交暂停或终止研究报告。
8. 研究者完成临床研究，请提交结题报告。
9. 凡涉及中国人类遗传资源采集标本、收集数据等研究项目，必须获得中国人类遗传资源管理办公室批准后方可在本院开展研究。

医学伦理委员会
